# Cardiac compression due to a large peri-prosthetic aneurysm of the descending aorta

**DOI:** 10.11604/pamj.2021.38.376.28981

**Published:** 2021-04-18

**Authors:** Hassen Ibn Hadj Amor, Cyrine Aousji

**Affiliations:** 1 Department of Cardiology, Taher Sfar Hospital, Mahdia, Tunisia

**Keywords:** Aortic aneurysm, aortic stent grafting, cardiac compression

## Image in medicine

A 77-year-old hypertensive man was admitted in cardiology department with acute chest pain and dyspnea. In his past medical history, he underwent endovascular aortic repair with stent grafting for a DeBakey III aortic dissection at the age of 64. His physical examination as well as a 12-lead electrocardiogram were unremarkable. Laboratory tests showed normal troponin levels. Renal function tests were normal. Hemoglobin count was 8.9 g/dL. Echocardiography revealed a preserved left ventricular function, a mild pericardial effusion and most importantly a large peri-prosthetic aneurysm with partial semi-circumferential thrombosis, compressing the left ventricle and the left atrium. Computed tomography (CT) angiography of the chest showed the same huge, partially thrombosed aneurysm of the descending aorta with a mass effect on the neighboring mediastinal structures. The course was marked by the worsening of symptoms and the increase in the size of the aneurysm. Based on these findings, the patient was referred to urgent surgery. During the operation, the patient passed away due to a fatal bleeding.

**Figure 1:**
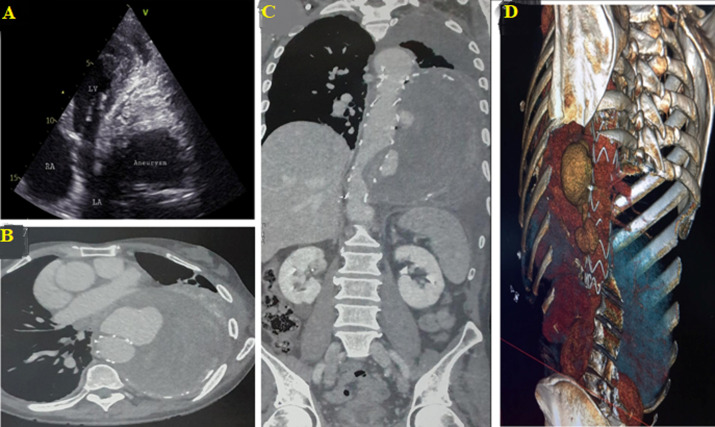
A) apical 4 chamber two-dimensional transthoracic echocardiogram showing compression of left cardiac chambers by descending thoracic aneurysm, with mural thrombus; B) contrast enhanced transversal computed tomographic imaging revealed eccentric saccular aneurysm of size 182 x 127 x 80 with mural thrombus arising from the arch and descending thoracic aorta, with compression of trachea, oesophagus and left cardiac chambers; C) contrast enhanced sagittal computed tomographic imaging revealed eccentric saccular aneurysm of size 182 x 127 x 80 with mural thrombus arising from the arch and descending thoracic aorta; D) three-dimensional abdominal angiographic CT scan showing peri-prosthetic descending aortic aneurysm

